# Elucidating the Role of Lipid-Metabolism-Related Signal Transduction and Inhibitors in Skin Cancer

**DOI:** 10.3390/metabo14060309

**Published:** 2024-05-28

**Authors:** Eunjin Kook, Do-Hee Kim

**Affiliations:** Department of Chemistry, Kyonggi University, Suwon 16227, Gyeonggi-do, Republic of Korea

**Keywords:** melanoma, lipid metabolism, skin cancer, resistance, small molecule inhibitors

## Abstract

Lipids, as multifunctional molecules, play a crucial role in a variety of cellular processes. These include regulating membrane glycoprotein functions, controlling membrane trafficking, influencing apoptotic pathways, and affecting drug transport. In addition, lipid metabolites can alter the surrounding microenvironment in ways that might encourage tumor progression. The reprogramming of lipid metabolism is pivotal in promoting tumorigenesis and cancer progression, with tumors often displaying significant changes in lipid profiles. This review concentrates on the essential factors that drive lipid metabolic reprogramming, which contributes to the advancement and drug resistance in melanoma. Moreover, we discuss recent advances and current therapeutic strategies that employ small-molecule inhibitors to target lipid metabolism in skin cancers, particularly those associated with inflammation and melanoma.

## 1. Introduction

In 2011, Hanahan and Weinberg recognized the deregulation of cellular metabolism as a fundamental hallmark of cancer cells, characterized by disruptions in multiple bioenergetic pathways, including those for glucose, amino acids, and lipids [1]. Among these metabolic agents, lipids are multifunctional molecules that significantly influence various cellular processes, including the regulation of membrane glycoprotein function, control of membrane trafficking, apoptotic pathways, and drug transport [2,3,4,5,6,7]. In addition, research has shown that lipid metabolites can influence the surrounding microenvironment in ways that may promote tumor progression [8,9,10].

Due to their uncontrolled growth and proliferation, cancer cells must alter metabolic processes to maintain these characteristics. These alterations in cellular metabolism result from the increased activity of oncogenes and the decreased activity of tumor suppressor genes. Tumorigenesis relies on these metabolic modifications, which arise either directly or indirectly from mutations in oncogenes. Cancer cells exploit this metabolic reprogramming to produce essential macromolecules and metabolites [11,12,13]. Moreover, cancer cells must adapt their metabolic processes to manage the challenging conditions of tumor microenvironments, which are marked by low oxygen levels, elevated oxidative stress, acidity, and scarce nutrients [14]. To adapt to hypoxia and nutrient shortages, tumor cells modify their metabolic pathways to meet their requirements for energy production, biosynthesis, and redox balance [15]. These disruptions aid in the metabolic adaptation of cancer cells, which, coupled with alterations in the tumor microenvironment, promote tumor progression [12,16,17]. Specifically, the reprogramming of lipid metabolism plays a central role in promoting tumorigenesis and cancer progression, with tumors often exhibiting significant alterations in lipid profiles [18].

Recent studies have highlighted lipid metabolic reprogramming as an adaptive shift in cellular processes under metabolic stress induced by glucose scarcity and a hypoxic environment [16,17,19]. Tumor cells alter various lipid metabolic processes, including De Novo lipogenesis, lipid transport, hydrolysis, and lipid oxidation, thereby increasing lipid levels and disrupting homeostasis. These changes support cell proliferation, survival, invasion, and metastasis [20,21,22,23]. Interestingly, it has been proposed that lipid metabolism is a significant hallmark in melanoma and inflammation-associated skin cancer [24,25,26]. Nonetheless, the investigation of the link between lipid metabolism and skin cancer remains nascent, underscoring the necessity for more thorough research.

In this review, we focus on the critical elements that drive lipid metabolic reprogramming, which enhances progression and drug resistance in melanoma. Additionally, we explore recent developments and current therapeutic approaches that use small-molecule inhibitors to target lipid metabolism in skin cancers associated with inflammation and melanoma.

## 2. Signaling Pathways and Target Proteins Associated with Lipid Metabolism in Skin Cancer

Dysregulated lipid metabolism in cancer cells is a key factor contributing to drug resistance and malignant behavior in melanoma. Changes in the lipid metabolism of melanoma cells can alter the composition and physical properties of their cell membranes, potentially initiating or perpetuating treatment resistance [24,27]. In this section, we examine various signaling mechanisms that regulate lipid metabolism within the scope of skin cancer. Table 1 presents the effects of regulating target proteins that are related to lipid metabolism on the progression of melanoma.

### 2.1. PI3K/AKT/mTOR Signaling

The phosphatidylinositol 3-kinase (PI3K)/AKT and mammalian target of rapamycin (mTOR) signaling pathways, which are often activated in melanoma, play a crucial role in conferring resistance to targeted therapy, immunotherapy, and chemotherapy [28,29,30,31]. PI3Ks, a unique and conserved group of intracellular lipid kinases, phosphorylate the 3′-hydroxyl group on phosphatidylinositol [32]. This phosphorylation triggers the activation of various intracellular signaling pathways that govern cell metabolism and survival [33]. Focusing specifically on phosphatidyl inositol phosphates (PIPs) as targets, the activated PI3K catalyzes the conversion of PIP2 to PIP3, thus regulating processes such as membrane trafficking, cell growth, metabolism, and migration [33,34]. PI3Kδ expression is especially upregulated in the skin of psoriatic patients and in proliferating psoriatic keratinocytes stimulated by proinflammatory cytokines such as IL-22 and TNF-α [35]. Inhibiting PI3Kδ activity with seletalisib led to a significant decrease in the TNF-α-induced phosphorylation of Phosphoinoisitide dependent kinase 1 (PDK1) and abrogated Akt phosphorylation. This led to a decrease in the expression of inflammatory genes, including CXCL8, CCL2, CCL5, HBD-2, and GM-CSF [35]. Additionally, seletalisib inhibits the proliferation, migration, and differentiation of psoriatic keratinocytes induced by IL-22 [35]. Moreover, administering seletalisib topically for 5 days along with imiquimod application improved the histological changes in psoriasiform murine skin manifestations. This treatment reduced acanthosis and scale thickness by approximately 50% and significantly decreased the infiltration of inflammatory cells in the dermis by about 35% [35].

mTOR, a key molecule in the downstream PI3K/AKT pathway, substantially influences cell proliferation and apoptosis [36,37,38]. The overall incidence of somatic non-synonymous mutations in mTOR was 10.4%. These mutations in mTOR can be recognized as indicators of poor prognosis in melanoma [39]. The Akt pathway inhibitors LY294002, wortmannin, and rapamycin, when used in combination with either cisplatin or temozolomide, not only markedly induce apoptosis in metastatic melanoma cell lines but also abrogate the invasiveness of melanoma cells in organotypic cultures [29]. These effects are linked to the near complete eradication of the antiapoptotic Bcl-2 family protein Mcl-1 [29]. Rapamycin-insensitive companion of mTOR (Rictor) has been recognized as a new interacting partner that plays an essential role in the kinase function of mTORC2 [40,41]. Rictor knockout mice exhibit a transient ichthyosis-like phenotype, which arises from the disrupted De Novo synthesis of epidermal lipids, altered structure of lipid lamellae, and improper filaggrin processing, all of which are regulated by mTORC2 [42]. In prenatal Rictor knockout mice, there is a noticeable increase in the expression of stress-responsive genes in the epidermis. This includes a significant elevation in genes related to epidermal barrier stress, intermediate filament genes, and genes activated by UVB radiation [42].

**Figure 1:**
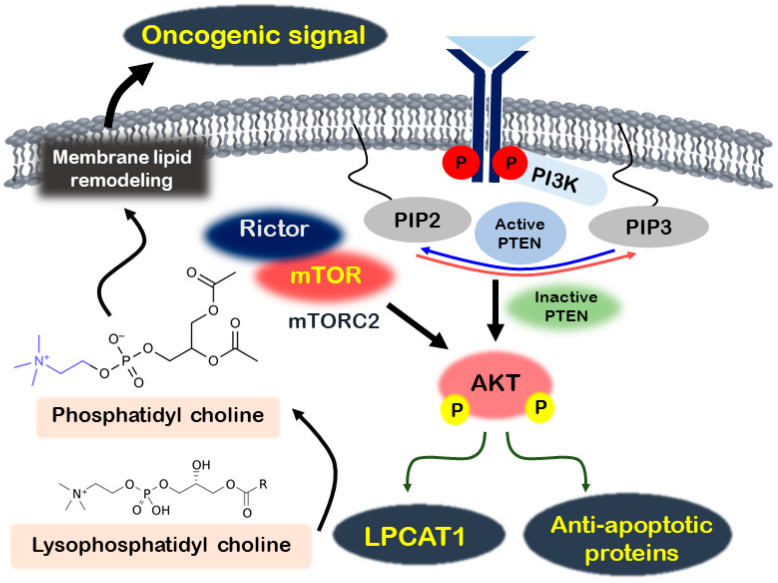
Regulation of melanoma through the Akt signaling activation mechanism. In melanoma, activated PI3K stimulates Akt, which then promotes the expression of antiapoptotic proteins and LPCAT1. LPCAT1 transforms lysophosphatidyl choline into phosphatidyl choline, activating oncogenic signals by remodeling membrane lipids. Additionally, the mTORC2 complex, comprising mTOR and Rictor, further enhances Akt phosphorylation.

Recent research has highlighted the role of Akt in influencing lipid composition changes in melanoma [43,44]. Lysophosphatidylcholine acyltransferase 1 (LPCAT1), an essential enzyme in lipid remodeling that converts lysophosphatidylcholine into phosphatidylcholine, has been shown to promote melanoma proliferation. LPCAT1 promotes melanoma cell growth in an Akt-dependent manner, and its knockdown leads to cell cycle arrest at the G1/S transition [43]. Additionally, the lipid phosphatase activity of phosphatase and tensin homolog (PTEN) suppresses fos-related antigen 1 (FRA1) expression through the AKT/mTOR signaling pathway, thereby reducing melanoma cell proliferation, invasion, and tumor growth [44]. Figure 1 presents a summary of the regulatory mechanisms involved in the PI3K/AKT/mTOR signaling pathway in melanoma.

### 2.2. Fatty Acid Synthase (FASN)

FASN, a multifunctional anabolic enzyme, catalyzes the synthesis of endogenous fatty acids from the small carbon precursors acetyl-CoA and malonyl-CoA [45]. FASN upregulation is a widespread phenotypic change observed in the majority of human malignancies, including colon, liver, and ovarian cancers [46,47,48,49]. In addition, patients with cutaneous melanomas that display high FASN levels have an increased risk of metastasis and disease recurrence [50]. Moreover, continuous FASN expression in B-Raf mutant patient-derived xenografts enhances lipogenesis, leading to the production of saturated and monounsaturated fatty acids that are integrated into cell membranes, thereby increasing membrane saturation [51]. Consequently, this process enhances the sensitivity of the cancer cells to reactive oxygen species (ROS) [51].

Dei Cas et al. reported an association between resistance to B-Raf/MEK inhibitors in melanoma and alterations in lipid pathways. They observed that that expression levels of FASN and seladin-1/24-dehydrocholesterol reductase (DHCR24) are correlated with the disease state, which is linked to differences in plasma cholesterol and triglyceride levels between patients and control subjects [31]. Lipidomic analysis revealed elevated levels of dihydroceramides, ceramides, sphingosine, sphingosine-1-phosphate, dihydrosphingosine, sphingomyelins, ganglioside GM3, and a range of both saturated and unsaturated fatty acids [31]. DHCR24 is expressed at higher levels in melanoma metastases compared to primary tumors, and its elevated expression is associated with resistance to apoptosis [52]. Suppressing DHCR24 with the inhibitor U18666A enhanced the sensitivity of the melanoma cells to hydrogen peroxide, demonstrating the protective role of DHCR24 against oxidative stress [52].

Vascular endothelial growth factor (VEGF), an antiangiogenic factor, is often underexpressed in metastatic melanomas and serves as a marker of their metastatic potential [53]. Capacity of orlistat to inhibit angiogenesis, demonstrated through its impact on VEGFA_165b_, is evidenced by the reduced growth of endothelial cells and hindered development of capillary-like structures in vitro [54]. Additionally, conditioned media from orlistat-treated human cancer cells (SK-MEL-25 and SCC-9) also reduce the proliferation of HUVECs [54]. Moreover, in the myoma organotypic invasion assay, SCC-9 ZsGreen LN-1 cells, a derivative of head and neck squamous cell carcinoma, exhibit increased invasiveness compared to their original SCC-9 counterparts [55]. Treatment with orlistat and FASN-targeted siRNAs significantly boosts the production of VEGFA_165b_ in these cells. Orlistat not only hinders the migration of SCC-9 ZsGreen LN-1 cells but also reduces the size of primary tumors and the incidence of lymph node metastases in orthotopic oral tongue squamous cell carcinoma [55].

Diet-induced obesity exacerbates melanoma progression in male C57BL/6J mice, which is associated with the elevated expression of Caveolin-1 (Cav-1) and FASN in tumors from mice on a high-fat diet [56]. In melanoma cells, Cav-1 interacts with FASN, and elevated levels of both Cav-1 and FASN, along with phosphorylated Akt, promote cell proliferation [56]. Using FASN inhibitors or specific siRNAs targeting FASN reduces FASN protein levels, significantly decreases proliferation, and lowers Cav-1 and phosphorylated Akt levels. The palmitoylation of Cav-1 may be a crucial mechanism for its stabilization at the protein level in melanoma cells [56]. Additionally, Simiczyjew and colleagues observed that adipocytes associated with melanoma exhibit reduced levels of adipogenesis markers and a significant decrease in lipid droplets, indicating their transformation into fibroblast-like cells. Furthermore, melanoma cells prompt a decrease in lipid content within adipocytes, likely due to heightened delipidation or diminished lipid synthesis [57].

Recently, the enzyme BCKDHA, known as branched-chain keto acid dehydrogenase E1 subunit alpha, which is involved in branched-chain amino acid metabolism, has been found to regulate the expression of FASN and ATP-citrate lyase (ACLY) in melanoma. This regulation enhances proliferation, invasion, migration, and tumor growth in vivo [58]. The expression of branched-chain amino acid transaminase 2 (BCAT2) also affects the levels of FASN and ACLY, promoting De Novo lipogenesis and facilitating melanoma progression. This progression is driven by the epigenetic modification of both FASN and ACLY via p300-dependent histone acetylation [58]. Based on these reports, FASN is widely recognized as a promising therapeutic target for melanoma and cutaneous squamous cell carcinoma.

### 2.3. Sterol Regulatory-Element-Binding Proteins (SREBPs)

SREBPs belong to the basic helix–loop–helix–leucine zipper family of transcription factors, and they activate the expression of genes that govern the biosynthesis of cholesterol and fatty acids [59]. In human melanoma cells, the expression of ganglioside GD3 regulates SREBP activity and cholesterol biosynthesis via the PI3K-Akt-mTORC1 signaling pathway cells [60]. Interestingly, this pathway exhibits a positive feedback loop, where enhanced SREBP via PI3K-Akt-mTORC1 further amplifies Akt signaling in GD3-expressing human melanoma cells. Blocking the SREBP pathway with 25-hydroxycholesterol and cholesterol synthesis inhibitors reduces Akt activation in lipid rafts and limits the growth of human melanoma cells [60]. In addition, the administration of compactin, an 3-hydroxy-3-methylglutaryl coenzyme A (HMG-CoA) reductase inhibitor, or 25-hydroxycholesterol significantly reduces melanoma cell growth in xenograft models [60].

Cancer stem cells derived from melanospheres exhibit an increased uptake of lipids compared to their differentiating counterparts, along with the robust expression of lipogenic factors such as SREBP-1 and peroxisome proliferator-activated receptor-γ (PPARγ) [61]. Additionally, an increase in autophagic flux, measured by microtubule-associated protein 1A/1B-Light Chain 3 (LC3) levels with and without the use of bafilomycin A1, is associated with a decrease in lipid droplet storage as melanoma stem cells differentiate [61]. In melanoma cells, levels of LC3 increase during progressive differentiation, indicating enhanced autophagic flux. These melanosphere-derived melanoma cells display a lipid-storing phenotype [61]. Moreover, the stemness marker CD133 is more highly expressed in cancer stem cells derived from melanospheres than in their differentiating counterparts [61].

Eukaryotic elongation factor-2 kinase (eEF2K) plays a critical role in melanoma cells, affecting both protein translation and cholesterol metabolism, particularly by regulating the translation of mRNA for SREBP-2, a key factor in cholesterol biosynthesis [62]. Targeting eEF2K through siRNA or the specific inhibitor NH125 not only impairs key elements of the cholesterol pathway but also suppresses tumor growth by diminishing the levels of SREBP-2 and its downstream targets. These targets include low-density lipoprotein receptor (LDL-R), crucial for cholesterol uptake, and HMG-CoA reductase, the rate-limiting enzyme in cholesterol biosynthesis [62]. This reduction impedes melanoma cell proliferation and overall cholesterol metabolism, crucial for cell survival, by modulating the phosphorylation of eEF2 [62].

**Figure 2:**
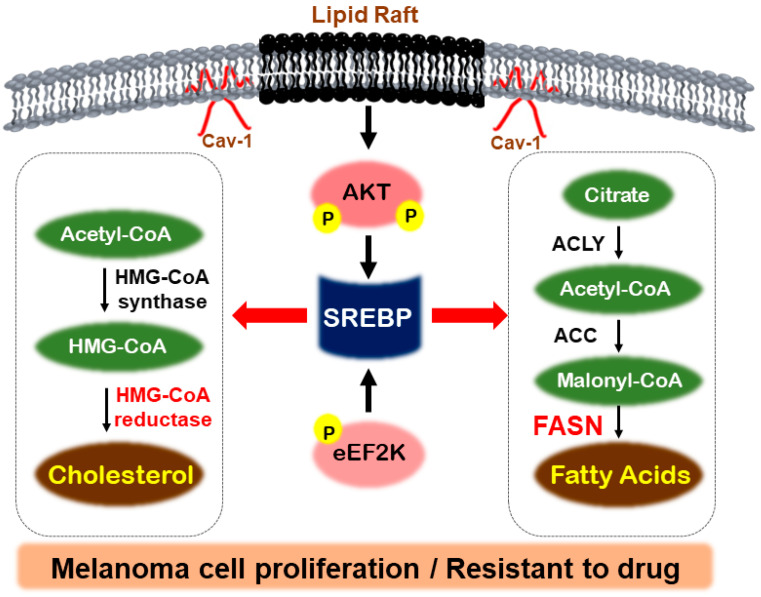
Regulatory mechanisms by SREBP in melanoma. In melanoma, lipid rafts, activated by Caveolin-1, can induce phosphorylation of Akt and promote SREBP activity. Additionally, eEF2K phosphorylation can activate SREBP, which then stimulates fatty acid uptake and cholesterol synthesis through FASN and HMG-CoA reductase. This activation contributes to the proliferation of melanoma cells and their resistance to drugs.

Ganglioside GD3 facilitates the tyrosine phosphorylation of focal adhesion kinase and paxillin, which in turn amplifies the growth and invasion of malignant melanoma cells by orchestrating the assembly of integrins within glycolipid-enriched microdomains [63,64]. In addition, GD3 expression initiates the activation of both SREBP-1 and SREBP-2, leading to an increased expression of HMG-CoA reductase and enhanced cholesterol biosynthesis in melanoma cells. This activation of the SREBP pathway occurs independently of oncogenic B-Raf mutations and is regulated by PI3K-Akt-mTORC1 signaling [60]. Figure 2 provides an overview of the signaling mechanisms associated with SREBP and FASN.

### 2.4. Peroxisome Proliferator-Activated Receptor-Gamma Coactivator (PGC)-1α

PGC-1α is known as a member of a superfamily of transcriptional coregulators that work alongside transcription factors [65,66]. It plays a role in controlling several activities related to cancer development, including the catabolism of glucose and fatty acids, as well as enhancing gluconeogenesis and lipogenesis [67,68,69]. The function of PGC-1α in cancer remains a subject of debate [70]. Reducing PGC-1α levels leads to an increase in integrin transcripts in melanoma cells, while overexpressing it in these cells through adenovirus infection suppresses metastasis. This suppression occurs by activating the inhibitor of DNA-binding protein 2 (ID2) and inhibiting the transcription factor 4 (TCF4)-mediated gene transcription [71]. Liang et al. recently found that cells resistant to B-Raf inhibitors, characterized by lower levels of PGC-1α, display increased integrin-FAK signaling and improved survival signals when they detach from the extracellular matrix, which contributes to their heightened metastatic potential [72]. Resistance to MEK inhibitors in melanoma cells harboring B-Raf or N-Ras mutations leads to increased expression of transcription factor microphthalmia-associated transcription factor (MITF), which subsequently elevated levels of PGC-1α [73]. Treatment of AZD8055, a selective inhibitor of mTOR kinase, suppressed the expression of both PGC-1α and MITF, as well as their promoter activities [73]. However, melanoma cells positive for PGC-1α exhibit increased capacities for mitochondrial energy metabolism and ROS detoxification, which enable survival under conditions of oxidative stress [74]. There was a notable decrease in tumor size when PGC-1α was depleted, indicating its critical role in tumor progression [74]. The precise mechanisms underlying the effects of PGC-1α remain largely unclear and warrant further investigation.

**Table 1:** Effects of regulating target proteins related to lipid metabolism in melanoma.

Target Proteins	Effects on Melanoma Cells	Reference
PI3Kδ ↓	IL-22-induced psoriatic keratinocyte proliferation ↓	[35]
mTOR ↓	Invasiveness of melanoma cells in organotypic cultures ↓	[35]
LPCAT1 ↑	Akt-dependent cell growth ↑	[43]
PTEN ↓	Proliferation of melanoma cells through FRA1 expression ↓	[44]
FASN ↑	Lipogenesis in B-Raf mutant patient-derived xenograft ↑	[51]
FASN ↓	Invasiveness through VEGFA_165b_ production ↓	[55]
FASN ↓	Proliferation of melanoma cells through Cav-1 palmitoylation ↓	[56]
DHCR24 ↓	Sensitivity of melanoma cells against oxidative stress ↑	[52]
Ganglioside GD3 ↑	Growth and invasion through SREBP activation ↑	[52]
PGC-1α ↓	Metastasis through integrin expression ↑	[71]
PGC-1α ↑	Mitochondrial energy metabolism and ROS detoxification ↑	[74]

## 3. Therapeutic Inhibitors Targeting Lipid Metabolism in Melanoma

Numerous drugs targeting lipid metabolic reprogramming have been explored and are expected to become strategic therapies for tumors. These approaches focus on the metabolic complexity by targeting key proteins within relevant signaling pathways. Table 2 presents the effects of regulating small-molecule inhibitors of lipid metabolism in melanoma.

### 3.1. Orlistat

Orlistat, an FDA-approved drug used for obesity, effectively inhibits pancreatic and gastric lipases by targeting the thioesterase domain of FASN, thus exerting its therapeutic effects. Orlistat is a lipase inhibitor that suppresses fat-metabolizing enzymes [75]. It has been reported that the inhibition of FASN activity by orlistat treatment not only reduces cell proliferation in mouse metastatic melanoma B6-F10 cells but also restricts their metastatic spread when injected into the peritoneal cavity of C57BL/6 mice [76,77]. Using orlistat to inhibit FASN activity in B16-F10 mouse melanoma cells triggers the intrinsic apoptosis pathway, characterized by cytochrome c release and the activation of caspase-9 and -3, and it is accompanied by increased ROS production and elevated cytosolic calcium levels [78]. Moreover, orlistat not only reduces the size of lymph node metastases in B16-F10 cells intradermally injected into the ears of C57BL/6 mice but also increases the permeability of lymphatic vessels. Conditioned culture media from B16-F10 cells treated with orlistat lead to a reduction in the number of filopodia-like extensions in lymphatic endothelial cells. This reduction is associated with decreased VEGFR-3 expression and promotes apoptosis [79]. Orlistat not only reduces the number of regulatory T cells (Tregs) and decreases the incidence of spontaneous B16-F10 melanoma metastasis but it also enhances the populations of CD80/CD86 and IL-12-positive dendritic cells, granzyme B/NKG2D-positive NK cells, and perforin/granzyme-B-positive CD8 T lymphocytes in primary tumors [80]. Additionally, it boosts nitric oxide production in peripheral neutrophils, which contributes to the effective elimination of cancer cells [80].

B-Raf inhibitor PLX4032-resistant LM16R melanoma cells exhibit reduced FASN levels compared to their sensitive counterparts, notably in LM16 cells. The treatment of orlistat targeting FASN in melanoma LM16R cells leads to the upregulation of DHCR24, activating compensatory pathways that support drug-resistant growth [81]. These resistant cells, noted for their enhanced migration and invasiveness, exhibit increased sensitivity to the B-Raf inhibitor PLX4032 following orlistat treatment [81]. The disruption of genes related to lipid metabolism underlies resistance to B-Raf inhibitors, presenting an opportunity for targeted therapy via FASN inhibition [81]. The combination of the B-Raf inhibitor PLX4032 with inhibitors of lipogenic enzymes may lead to advantageous drug interactions, especially in the treatment of melanoma resistant to B-Raf inhibitors.

### 3.2. Cerulenin

Cerulenin, a potent natural FASN inhibitor, is an epoxide produced by the fungus *Cephalosporium caeruleus*. Similar to orlistat, cerulenin has also been reported to regulate melanoma growth and migration in B16-F10 mouse melanoma cells [78,79,81]. In addition, C75, formally recognized as 4-methylene-2-octyl-5-oxo-tetrahydro-furan-3-carboxylic acid, is a synthetic derivative of cerulenin. Both cerulenin and C75 hinder the growth of human melanoma A375 cells by disrupting the cell cycle and triggering apoptosis, which involves activating caspase-3 and is associated with the upregulation of p21 and the reduction in Bcl-xL and Mcl-1 [77]. Therefore, these reports also demonstrate that inhibiting FASN would be an effective strategy for preventing and slowing the growth of melanoma.

### 3.3. TVB-3644

TVB-3166, an orally bioavailable and selective small-molecule inhibitor of FASN, inhibits the De Novo synthesis of palmitate [82]. Despite their significant chemical similarity, TVB-3664 is eight to ten times more potent against human and mouse FASN than TVB-3166 [83]. Suppressing FASN activity decreases both tubulin palmitoylation and mRNA expression. These reductions in tubulin palmitoylation, coupled with changes in microtubule structure, enhance the sensitivity of tumor cells to the effects of taxanes [83]. TVB-3664 enhances the uptake of lipids, rich in ROS-labile polyunsaturated fatty acids (PUFAs), by cancer cells. This, in turn, increases the susceptibility of the polyunsaturated cell membranes to ROS [51]. Moreover, by leveraging this susceptibility and combining ROS inducers with mitogen-activated protein kinase (MAPK) and FASN inhibitors, the development of therapy resistance is significantly delayed [51].

### 3.4. Fatostatin

Fatostatin, also known as the diarylthiazole derivative, stands as the inaugural non-sterol-like synthetic molecule to impede the activation of SREBPs. It seems to inhibit the ER–Golgi translocation of SREBPs by binding to their escort protein, the SREBP cleavage-activating protein (SCAP), at a site distinct from the sterol-binding domain [84]. Persistent SREBP-1 activity perpetuates lipogenesis in melanoma cells resistant to B-Raf inhibitors. The pharmacological inhibition of SREBP-1 with fatostatin enhances the responsiveness of B-RafV600E-mutant, therapy-resistant melanoma to B-RafV600E inhibitors, both in vitro and in preclinical patient-derived xenograft models in vivo [85]. In addition, phospholipidomic analysis reveals that chemically inhibiting SREBP-1 results in a dose-dependent decrease in both monounsaturated and fully saturated phospholipid species, along with an increase in membrane polyunsaturation [85]. Moreover, suppressing SREBP-1 enhances the efficacy of vemurafenib in a preclinical PDX melanoma model. The inclusion of the antioxidant NAC restores cell proliferation when used alongside combined therapy [85].

It is known that dysregulated lipid metabolism affects T cell responses within tumors and overall tumor growth. Recently, Zhu et al. reported that fatostatin significantly suppresses the growth of transplanted B16 melanoma in mice by inhibiting SREBP-2-mediated lipid metabolism, particularly reducing cholesterol levels [86]. Additionally, it reduced intracellular cholesterol and mitigated X-box-binding-protein-1-mediated endoplasmic reticulum stress, which resulted in a decrease in Treg cells and reduced CD8+ T cell exhaustion in the tumor microenvironment (TME), thereby enhancing its antitumor effects [86]. Moreover, fatostatin markedly altered metabolic levels, notably impacting the metabolism of glycolysis, gluconeogenesis, and cysteine and methionine [86]. Fatostatin shows potential for clinical application across various cancer models, including melanoma.

### 3.5. Betulin/Betulinic Acid

Betulin, a pentacyclic triterpene and a derivative of plant pentacyclic triterpenes, binds directly to SCAP, inhibiting the cleavage and activation of SREBP-1 [87]. Similar to fatostatin, betulin has been shown to affect the resistance of melanoma cells to B-Raf inhibitors under the same experimental conditions [85]. Betulinic acid, produced through the oxidation of betulin, triggers programmed cell death in human melanoma UISO-Mel-1 cells and in cell lines derived from human cancer patients with metastatic melanomas [88,89]. Treatment with betulinic acid leads to the generation of ROS in these cells. Pretreating these cells with antioxidants blocks both programmed cell death and the phosphorylation of signaling molecules such as p38 and c-Jun N-terminal kinase (JNK) [89]. In addition, by repressing the expression of neutrophil gelatinase-associated lipocalin, betulinic acid exerts its antimetastatic potential in melanoma cells by reversing the epithelial–mesenchymal transition [90]. The potential of betulinic acid and its derivatives has been demonstrated in numerous preclinical studies using various experimental models [91].

**Table 2:** Effects of small-molecule inhibitors of lipid metabolism in melanoma.

Small-Molecule Inhibitors	Molecular Targets	Effects on Melanoma Cells	Ref.
Orlistat	■ Thioesterase domain of FASN	■ Reduced cell proliferation in mouse metastatic melanoma B6-F10 cells ■ Inhibition of metastatic spread in melanoma-cell-injected mouse model ■ Reduction of Treg population ■ Increased population of CD80/CD86, IL-12-positive dendritic cells, granzyme B/NKG2D-positive NK cells, perforin/granzyme-B-positive CD8 T lymphocytes	[76,77,78,80]
Cerulenin C75	■ FASN	■ Inhibition of melanoma growth and migration ■ Cell cycle arrest of A375 cells through p21 upregulation ■ Apoptosis through caspase-3 activation and reduction of Bcl-xL and MCl-1	[77,78,79,81]
TVB-3644	■ FASN	■ Inhibition of palmitate synthesis ■ Suppression of tubulin palmitoylation ■ Uptake of lipids rich in polyunsaturated fatty acids	[51,83].
Fatostatin	■ Directly bind to SCAP ■ Inhibit SREBP activation	■ Enhancement of responsiveness of melanoma cells resistant to B-Raf inhibitor ■ Suppression of growth of transplanted B16 melanoma in mice	[85,86].
Betulin/Betulinic acid	■ Directly bind to SCAP ■ Inhibit SREBP activation	■ Apoptosis of metastatic melanoma cells and B-Raf-inhibitor-resistant melanoma cells ■ Blocking the phosphorylation of JNK and p38	[85,89]

## 4. Lipid Metabolic Dynamics in the Melanoma Microenvironment

It has been shown that melanoma cells induce metabolic shifts in adipocytes, leading to lipolysis and the subsequent release of fatty acids [92,93]. Adipocytes play a role in the emergence of drug resistance in melanoma by facilitating the acquisition of resistance to melanoma treatments. Lipids present around the cancer cells can be directly absorbed by melanoma cells and used as an energy source for rapid growth [94]. Moreover, adipocytes affect melanoma cell invasion by prompting the production of oncogenic proteins such as cyclin D1 and cyclooxygenase 2 and by stimulating the Akt/mTOR pathway [95]. Furthermore, fat cells secrete proangiogenic factors like hepatocyte growth factor (HGF) and VEGF, which contribute to the vascular mimicry associated with melanoma [96].

Simiczyjew et al. demonstrated that adipocytes undergo delipidation when cocultured with melanoma cells, likely due to the uptake of lipids by the cancer cells [57]. Additionally, an elevation in perilipin 2 levels was observed in melanoma cells that were cocultured with adipocytes. To confirm the impact of adipocytes on lipid accumulation in melanoma cells, lipid droplet staining was conducted, which showed increased lipid levels in metastatic melanoma cells after coculturing with adipocytes [97]. Moreover, a rise in lipid content was recorded in metastatic melanoma cells after coculture with cancer-associated adipocytes [97], corroborating the findings by Kwan et al., who noted higher levels of fatty acids, particularly palmitoleic acid, in melanoma cells incubated with adipocytes [98].

Melanoma cells with higher levels of membrane saturation show decreased sensitivity to oxidative stress induced by targeted therapeutic agents such as B-Raf/MEK inhibitors [99]. Membrane microdomains, often called rafts, are rich in cholesterol, which enhances the adaptive resistance of melanoma cells to MAPK pathway inhibitors [100]. Reducing cholesterol not only obstructs the feedback activation of phosphoproteomic signaling but also increases the cytotoxic effects of MAPK pathway inhibitors. These rafts play a role in increasing resistance to apoptosis in melanoma cells, indicating that targeting these specific areas of the membrane might be a potent strategy for melanoma treatment [101]. Alicea et al. found that blocking crucial signaling pathways in lipid metabolism, such as fatty acid transport protein, can decrease lipid accumulation in melanoma cells and curtail their mitochondrial metabolic energy supply. This strategy has been proven to counteract B-Raf/MEK inhibitor resistance in mouse models [5]. Circulating tumor cells from melanoma patients show elevated lipogenic activities, such as SREBP activation, fatty acid metabolism, adipogenesis, and cholesterol regulation. These cells also engage more intensively in iron-related pathways, including oxidative phosphorylation, ferroptosis, and iron ion regulation, all verified by transcriptomic analysis [102]. Transferrin, a transcriptional target of the lipogenic regulator SREBP-2, is critical for iron trafficking and metabolism. Its expression helps manage the intracellular labile free iron pool, affecting ROS and lipid peroxidation, which determine cellular susceptibility to ferroptosis [102]. Knocking down endogenous transferrin impairs tumor formation by melanoma circulating tumor cells; however, this defect in tumorigenesis can be partially offset by lipophilic antioxidants such as ferrostatin-1 and vitamin E [102]. SREBP-2 directly promotes transferrin expression, which lowers intracellular iron levels, ROS stress, and lipid peroxidation, thereby inhibiting ferroptosis to boost the survival and drug resistance of circulating tumor cells [102]. In addition, increased lipogenesis driven by SREBP, including the expression of its target glutathione peroxidase 4, further counters ferroptosis. Overall, the reduction in transferrin significantly obstructs tumor formation [102].

## 5. Concluding Remarks and Perspectives

Exploring lipid metabolism as a target may unveil new therapeutic avenues in cancer treatment, as highlighted by referenced studies. This strategy focuses on altering the reprogramming factors that promote melanoma progression. Alterations in lipid metabolism, such as enhanced lipogenesis, along with changes in lipid transport and oxidation, support key oncogenic processes such as cell proliferation, survival, invasion, and metastasis. These metabolic changes also affect the composition and physical properties of cell membranes in melanoma cells, potentially triggering or exacerbating resistance to treatment. Considering the varied influences on lipid metabolism, it is critical to conduct a comprehensive analysis of its expression patterns and interactions with the microenvironment to effectively manage skin cancers, including melanoma. Although general articles already summarize the importance of lipid metabolism in skin cancer models [103,104], we anticipate that this review article, together with an introduction to recent updates in the field, will offer valuable insights for further research.

In a recent study, Zhang et al. demonstrate that activating PPARγ, the canonical activator of lipid uptake and adipogenesis, with thiazolidinediones enhances the efficacy of immunotherapy in mouse melanoma [105]. This could act as a prognostic marker associated with enhanced immunotherapy effectiveness in humans with melanoma [105]. The TME mediates crucial phenotypic changes that promote melanoma metastasis, constituting a complex network where malignant cells not only interact among themselves but also with stromal and immune cells. Therefore, it is essential to broaden our understanding of lipid metabolism to encompass the TME of melanoma.

Lipids are crucial not only in forming cellular membranes but also as bioactive lipid mediators and key components of the skin barrier. Many of the microenvironmental cues that influence melanoma progression remain poorly understood. Extensive research has been conducted on the link between glucose metabolism and cancer; however, investigations into lipid metabolism are still in the early stages, suggesting a critical need for additional research and development.

## Data Availability

No new data were created or analyzed in this study. Data sharing is not applicable to this article.
